# Revealing the immune landscape of menstrual blood: unlocking insights into activation, exhaustion, and mitochondrial mass for reproductive health

**DOI:** 10.1093/immhor/vlag013

**Published:** 2026-03-25

**Authors:** Oliver Richards, Megan Cotterell, Catherine A Thornton, April Rees

**Affiliations:** Institute of Life Science, Swansea University Medical School, Swansea, Wales, United Kingdom; Institute of Life Science, Swansea University Medical School, Swansea, Wales, United Kingdom; Institute of Life Science, Swansea University Medical School, Swansea, Wales, United Kingdom; Institute of Life Science, Swansea University Medical School, Swansea, Wales, United Kingdom

**Keywords:** endometriosis, immune cells, menstrual blood, PCOS, reproduction

## Abstract

Reproductive disorders such as endometriosis and polycystic ovary syndrome (PCOS) are increasingly recognized as immune-mediated conditions, yet their immunopathology remains poorly understood. Menstrual blood, a noninvasive and biologically relevant sample, offers a unique window into reproductive tract immunity but has been underutilized in this context. We optimized Cytek’s^®^ 25-color high-dimensional flow cytometry panel by incorporating a mitochondrial dye to investigate immune cell profiles in menstrual mononuclear cells (MMCs) from healthy individuals, and those with endometriosis or PCOS, in comparison with matched peripheral blood mononuclear cells (PBMCs). This enabled detailed assessment of 40 immune cell subsets and 546 immunological parameters, including markers of activation, exhaustion, migration, and mitochondrial content. MMCs displayed a distinct immune landscape compared to PBMCs, enriched with tissue-resident NK cells, macrophages, and dendritic cells, alongside changes in mitochondrial mass for various cell subsets and other markers such as PD-1. These findings support a metabolically active, tissue-adapted immune environment within menstrual fluid, representative of the reproductive tract. Exploratory analyses of MMCs from individuals with endometriosis or PCOS revealed disease-specific trends: for example, mitochondrial mass differed across Tregs, CD4 central memory cells, plasmablasts, and cDC1s, with endometriosis and PCOS exhibiting distinct patterns rather than a uniform “reproductive disorder” phenotype. Although these disease-associated findings did not consistently reach statistical significance due to the small cohort size, they demonstrate the potential of menstrual blood immunoprofiling to uncover biologically meaningful differences across diverse immune cell populations. Together, this study establishes menstrual fluid as a valuable, non-invasive sample for immunological assessment and a promising avenue for future biomarker discovery in reproductive disorders.

## Introduction

Reproductive disorders such as endometriosis and polycystic ovary syndrome (PCOS) have seen increased interest in recent years due to their increasing prevalence and recognition that they result in debilitating symptoms beyond infertility. However, our understanding of reproductive disorders is limited, and endometriosis, for example, has poor diagnostic and treatment options, with no cure available. Inflammation is a normal feature of the menstrual cycle with dysregulated inflammation characteristic of endometriosis^1^^,^^2^ and PCOS.^3^ Investigation then into the changes of the immune system in these disorders is crucial to improve our understanding and to identify novel diagnostic and therapeutic routes. These inflammatory changes are described predominantly within the uterus, which is difficult to sample, but parallel changes systemically offer the opportunity to address knowledge gaps in our understanding of disease mechanisms to uncover novel treatments.

Menstrual blood, historically considered a waste product, has gained attention in recent years as a valuable resource for scientific research. Its rich cellular and molecular composition offers insights into various physiological and pathological processes, particularly in reproductive health, and it includes endometrial cells, stromal and immune cells (including monocytes, macrophages, natural killer [NK] cells and T cells), cytokines, growth factors, and extracellular vesicles.^4^^,^^5^ The presence of various cell types and biomolecules makes menstrual blood a noninvasive source for studying endometrial biology and immune responses. Indeed, research has shown that collection of menstrual blood mononuclear cells (MMCs) using a menstrual cup is reliable and reproducible.^5^

**Figure 1:**
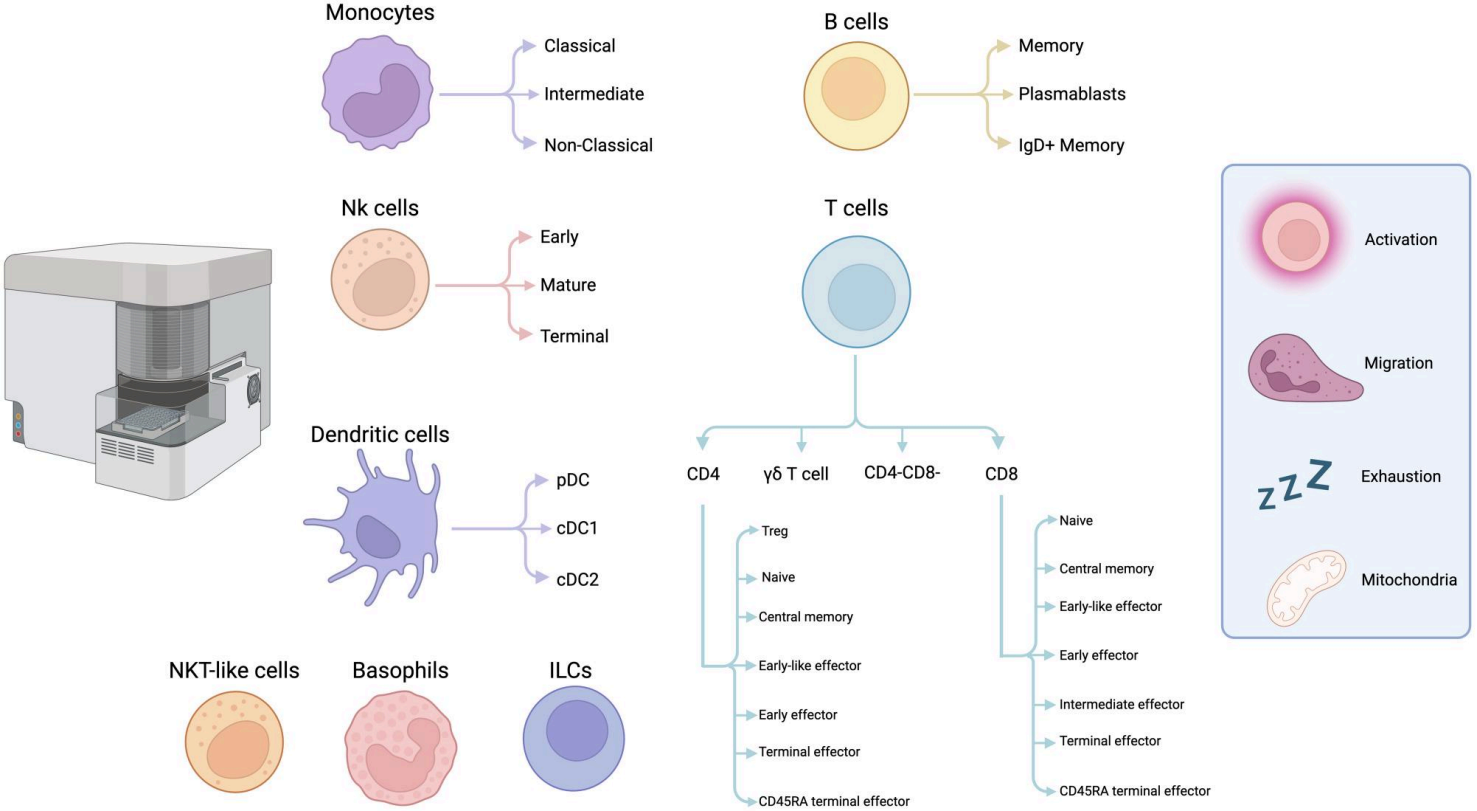
Schematic of the 27-color flow cytometry panel. A vast number of cell subsets are identified, with expression of markers covering activation, degranulation, exhaustion and mitochondria measurable. Created in BioRender. Rees, A. (2026) https://BioRender.com/h6qs1op.

However, the immune phenotype of the MMCs is still unknown in any depth. Here, we utilize high dimensional flow cytometry to profile the immune cells in healthy menstrual blood compared to matched peripheral blood, simultaneously analyzing markers for activation, exhaustion, and degranulation. As metabolism plays a key role in dictating immune cell fate and function,^6^ we incorporated a mitochondrial dye into Cytek’s^®^ established 25-color immune profiling panel in order to determine mitochondrial content, which would provide insights into the metabolic health of the cells,^7^^,^^8^ creating a 27-colour immunoprofiling panel (Fig. 1). This is a particularly novel application, as it provides this data for 40 different immune cell subsets, creating a total of 546 data points per sample. In addition, we show how the MMC landscape changes in endometriosis and PCOS in comparison to the healthy volunteers. This offers a unique perspective into the inflammatory changes in these disorders and opportunities for further investigation.

## Subjects and methods 

### Volunteers

Matched peripheral blood (collected into one 9 ml heparinized Vacuette ^TM^ [Greiner Bio-one, Frickenhausen, Germany) and menstrual blood (collected in a menstrual cup [Mooncup^®^, &SISTERS, UK]; volumes ranged from 4 ml to 15 ml) were donated from healthy volunteers in the community who either reported no symptoms of reproductive disorders, or self-reported diagnosis with endometriosis or PCOS. Participants were instructed on the appropriate use of a menstrual cup and the peripheral blood taken by venepuncture on their heaviest day of their period (day 1–3 of their cycle). Participants were required to transfer the menstrual blood from the menstrual cup to a sterile universal tube (with no anticoagulant) and to keep the sample refrigerated until it could be delivered to the lab within 4 h. All samples were collected with informed written consent and ethical approval was obtained from Swansea University Medical School’s (SUMS) research ethics sub-committee (RESC), project reference 2 2024 9064 8225.

### Menstrual blood processing

As menstrual blood is a viscous sample, 77 mg/ml of dithiothreitol (Pierce^TM^ DTT No-Weigh^TM^ Format [Thermo Scientific] in phosphate-buffered saline [PBS; Life Technologies]) was added to the menstrual sample, which was then agitated at room temperature for 30 mins until the sample dispersed. An equal volume of PBS was added to the menstrual blood before filtering through a 100 μm pluriStrainer (PluriSelect) to remove clots. DTT was used to reduce sample viscosity and enable efficient isolation of mononuclear cells.

### Mononuclear cell isolation

Mononuclear cells (MNCs) from peripheral blood (PBMCs) or processed menstrual blood (MMCs) were isolated by density gradient centrifugation using Lymphoprep^TM^ (Stem Cell Technologies, UK), and washed with RPMI 1640 and Glutamax (Life Technologies, Paisley, UK).

### Flow cytometry

Cells were analyzed using Cytek’s^®^ 25-color immunoprofiling assay^9^ (antibody information detailed in Table S1) with the additional investigation into mitochondrial mass using BioTracker 405 blue mitochondrial dye (final concentration 50 nM; Sigma-Aldrich) and ViaDye^TM^ Red fixable viability dye (Cytek^®^) to create a 27-color panel. Briefly, 2 × 10^6^ MNCs were washed with PBS containing 0.2% BSA and 0.05% sodium azide and pellets were resuspended with 100 µl of the 50 nm mitochondrial dye and 5 µl ViaDye^TM^ Red fixable viability dye and incubated at 37 °C with 5% CO_2_ for 30 min. Cells were washed again, and the remainder of the protocol is as described by Cytek^®^. Flow cytometry data were acquired using the Cytek^®^ Aurora (4 l V/B/YG/R) spectral flow cytometer and analysed using SpectroFlo^®^ (Version 3.3.0; Cytek^®^), where compensation was applied by unmixing with autofluorescence extraction to address any spectral overlap. Appropriate controls were used: unstained and single stains to correct for fluorescence spillover. Quality control (QC) beads (Cytek) were used daily to reduce intersession instrument variability.

### Data analysis

Uniform manifold approximation and projections (UMAPs) were created using the Spectre workflow R package (Ashhurst et al.;^10^ source code and instructions found at https://github.com/ImmuneDynamics/spectre), with samples first prepared in FlowJo (version 10.1; BD Biosciences) where the population of interest (single live CD45^+^ cells) were exported as raw value CSV files to be processed and analyzed using R (Version 2023.12.1, RStudio). Other packages used include FlowSOM^11^ for clustering the data, and the UMAP algorithm.^12^ In addition to this, heatmaps were created based on the clustering of cells identified and their markers.

Cell subsets (including further subsetting of the CD4 T cells etc), percentages (of parent and of total CD45^+^ cells) and their expression of the various markers (Mitochondria, HLA-DR, PD-1, CD11c, CD25, CD27, CD28, CD38, CD123, CD127, CCR7) were identified on SpectroFlo^®^ (gating strategy for identification of cell subsets in Figs. S3–5 and Table S2). Data were exported as csv files to be analyzed using R (packages include tibble,^13^ dplyr,^14^ tidyr^15^) approximately 546 data points were analyzed per sample. A Kolmogorov–Smirnov (K-S) test was used to check for normality for each data point across samples; a *t* test was performed if the K-S *P* value was >0.05, or a Mann–Whitney test was used if the *P* value was ≤0.05. A *P* value of <0.05 was determined to be significant.

## Results

### The profile of immune cells in menstrual blood is distinct from the peripheral blood and exhibits tissue-resident characteristics

**Figure 2:**
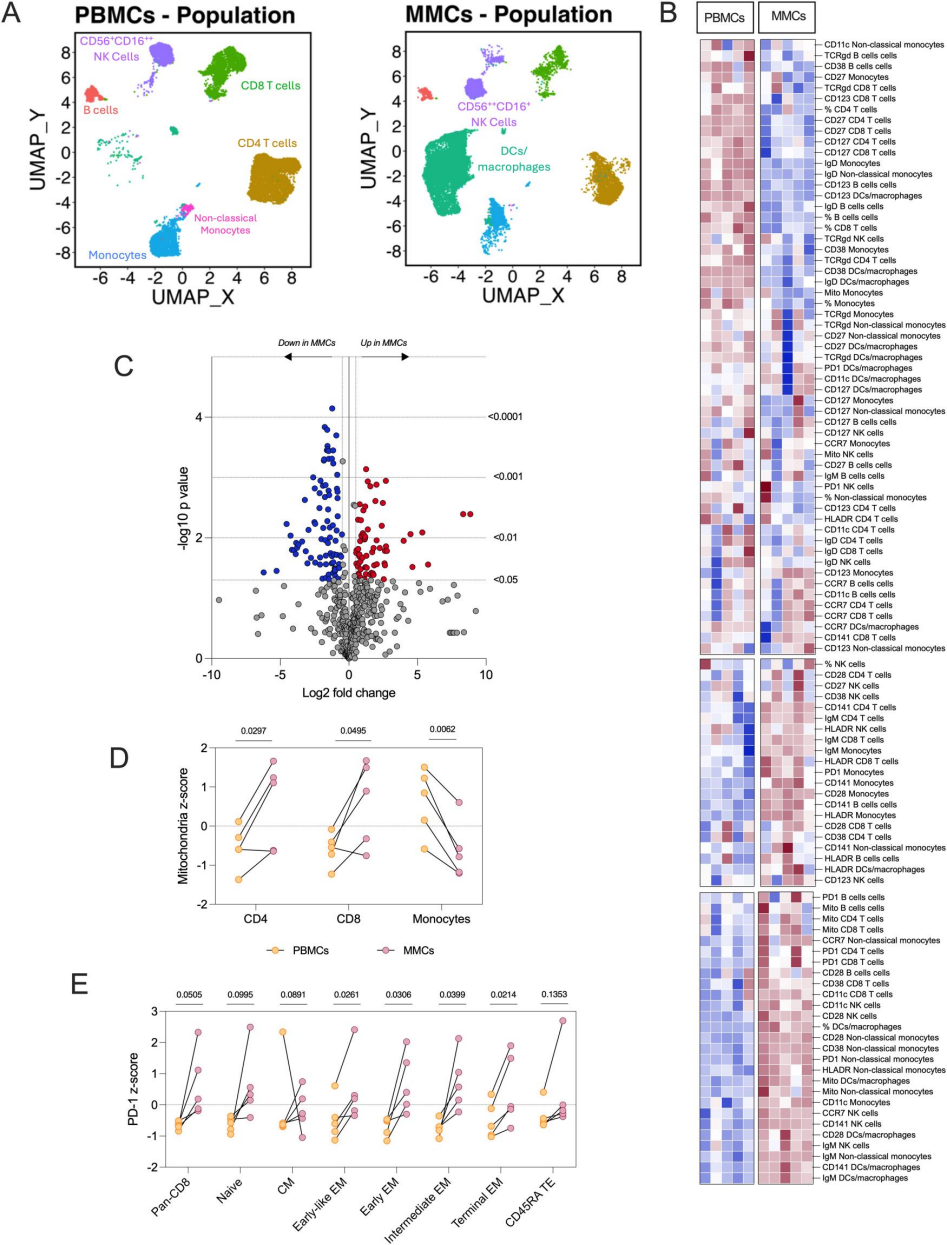
Exploration of the immune phenotype of menstrual blood mononuclear cells in comparison to matched peripheral blood mononuclear cells. Matched MMCs and PBMCs (*n* = 5) were isolated and analyzed with our 27-color panel. (A) UMAPs were created with R Studio, with unsupervised clustering based on the cellular expression of the markers measured for all pooled samples. UMAPs show the separated clusters for PBMCs and MMCs, with manual identification of cell populations. (B) Heatmap of the expression of various markers of the cells identified from the UMAP. (C) Cell populations and their markers were analyzed directly from the flow cytometry data; data points with a *P* value <0.05 and a Log2 fold change of <0.5 or >0.5 was considered significant. Data were further extracted and transformed into z-scores for (D) mitochondrial mass across CD4 and CD8 T cells, and monocytes, and (E) PD-1 expression across CD8 T cell subsets including: pan-CD8s, naive, central memory (CM), early-like effector memory (EM), early EM, intermediate EM, terminal EM, CD45RA^+^ terminal effectors (TE).

Matched peripheral blood and menstrual blood samples were collected from healthy volunteers on the same day, specifically during the peak of menstrual flow. To characterize the immune landscape of menstrual blood, we employed our 27-color immunoprofiling assay. Unbiased clustering analysis was performed using UMAP on live CD45^+^ populations, revealing 8 distinct immune cell clusters (Fig. 2A; Fig. S1A). Notably, a marked shift in NK cell phenotypes was observed: from CD56^dim^CD16^bright^ in PBMCs to CD56^bright^CD16^dim^ in menstrual mononuclear cells MMCs, indicating a greater representation of tissue-resident NK cells in MMCs. Additionally, dendritic cells (DCs) and macrophages were more prominent in MMCs, further distinguishing the immune profiles of these 2 compartments. Distinct differences were also observed in T cell and monocyte populations between PBMCs and MMCs. Based on the clustering, we subsequently visualized marker expression across different cell types (Fig. 2B).

To gain deeper insight, we analyzed the complete data set generated from the 27-color panel gating strategy (Figs. S3–5), yielding 546 individual data points per sample. Using bioinformatics tools, we calculated *P* values and log_2_ fold changes for each parameter (Fig. 2C), identifying 87 features significantly downregulated and 66 significantly upregulated in MMCs relative to PBMCs (Table S3). As it was not feasible or informative to present figures for all significant parameters, we selected representative markers that highlight key immunological differences between the two compartments and reflect the strengths of our extended 27-color panel. Given the incorporation of a mitochondrial mass dye into Cytek’s© 25-colour panel, we first extracted mitochondrial features of CD4 and CD8 T cells, and monocytes (Fig. 2D). CD4 and CD8 T cells in MMCs displayed notably higher mitochondrial mass compared with their PBMC counterparts, whereas monocytes showed reduced mitochondrial content. These findings suggest that T cells within menstrual fluid may rely more heavily on oxidative phosphorylation, consistent with a more tissue-resident or metabolically engaged phenotype. We also extracted expression of an immune checkpoint receptor PD-1 in CD8 T cells and their major subsets, highlighting the depth of the data (Fig. 2E). Although PD-1 tended to be higher across all CD8 subsets in MMCs, significant increases were observed specifically within the effector memory (EM) populations. This pattern indicates a more differentiated or chronically stimulated CD8 T cell compartment within menstrual fluid, further underscoring the distinct immune environment of this tissue-derived sample compared with peripheral blood.

### Menstrual blood offers insights into immune cell differences in reproductive disorders

**Figure 3:**
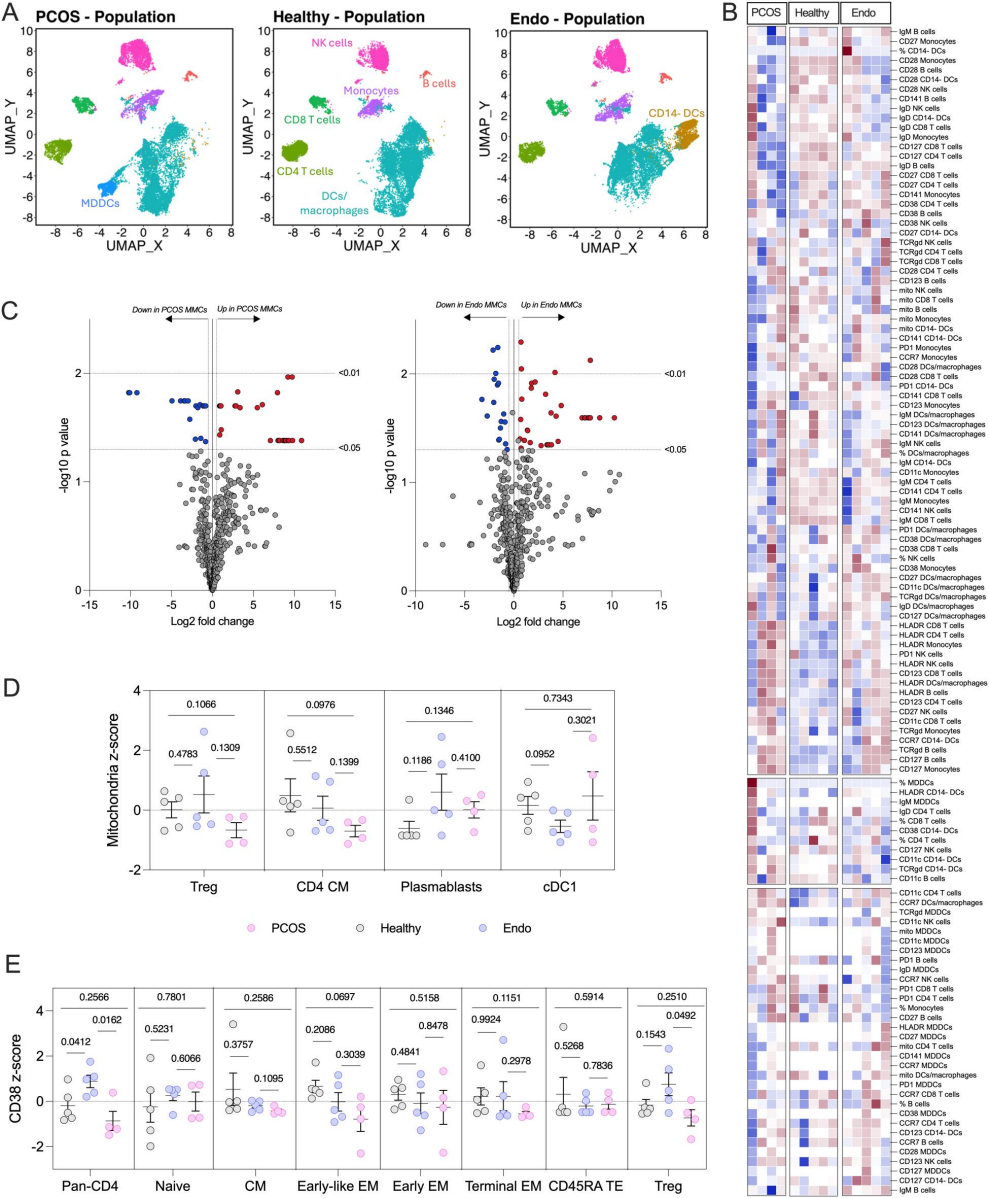
Utilizing menstrual blood mononuclear cells to distinguish between reproductive disorders. MMCs were isolated from healthy volunteers (*n* = 5), and volunteers with endometriosis (*n* = 5) or PCOS (*n* = 4) and analyzed with our 27-colour panel. (A) UMAPs were created with R Studio, with unsupervised clustering based on the cellular expression of the markers measured for all pooled samples. UMAPs show the separated clusters for PBMCs and MMCs, with manual identification of cell populations. (B) Heatmap of the expression of various markers of the cells identified from the UMAP. (C) Cell populations and their markers were analyzed directly from the flow cytometry data; data points with a *P* value <0.05 and a Log2 fold change of <0.5 or >0.5 was considered significant. Data was further extracted and transformed into z-scores for (D) mitochondrial mass across Treg, CD4 CM, plasmablasts, cDC1s, and (E) CD38 expression across CD4 T cell subsets including: pan-CD4s naive, CM, early-like EM, early EM, terminal EM, CD45RA+ TE, and Tregs.

Given that menstrual blood originates from the reproductive tract, it represents a biologically relevant and noninvasive sample for investigating reproductive disorders such as endometriosis and polycystic ovary syndrome (PCOS). Applying the same 27-color immunoprofiling assay and analytical approach described above, we observed distinct immune cell clustering patterns in menstrual blood from healthy volunteers compared to individuals with PCOS or endometriosis (Fig. 3A and B; Fig. S1B). Notably, monocyte-derived dendritic cells (MDDCs) emerged uniquely in PCOS samples, while CD14^−^ dendritic cells were more prominent in samples from individuals with endometriosis.

Further analysis of the 546 immunological features (Fig. 3C) revealed disease-specific alterations. In PCOS versus healthy controls, 31 features were significantly upregulated and 18 downregulated (Table S4). In the comparison between endometriosis and healthy controls, 35 features were upregulated and 17 downregulated (Table S5). Given the exploratory nature of the study and the breadth of parameters assessed, we selected representative markers to illustrate the differential immune signatures observed between conditions.

Mitochondrial mass was first assessed in selected immune cell subsets (Fig. 3D). Regulatory T cells (Tregs) showed a reduction in mitochondrial content in PCOS samples compared with both healthy individuals (*P *= 0.1066) and those with endometriosis (*P *= 0.1309), whereas no difference was evident between healthy and endometriosis samples (*P *= 0.4783). A similar pattern was observed for CD4 CM T cells, with mitochondrial mass trending lower in PCOS relative to healthy (*P *= 0.0976) and endometriosis (*P *= 0.1399) but with no difference between healthy and endometriosis groups (*P *= 0.5512). Plasmablasts showed increased mitochondrial mass in endometriosis compared with healthy (*P *= 0.1186), with a similarly elevated—though less variable—profile in PCOS (*P *= 0.1346). Differences between endometriosis and PCOS were not apparent (*P *= 0.4100). cDC1 populations demonstrated reduced mitochondrial mass in endometriosis compared with healthy samples (*P *= 0.0952), with PCOS displaying considerable heterogeneity that spanned both groups (healthy vs PCOS *P *= 0.7343; endometriosis vs PCOS *P *= 0.3021). Collectively, these data illustrate that mitochondrial alterations are not uniformly associated with “reproductive disorder” status but reflect disease-specific immune features.

To further explore disease-associated immune variation, we assessed CD38 expression across CD4 T cells and their major subsets (Fig. 3E). In pan-CD4 T cells, endometriosis samples showed higher CD38 expression than both healthy (*P *= 0.0412) and PCOS samples (*P *= 0.0162), whereas PCOS displayed a slight, non-significant reduction relative to healthy individuals (*P *= 0.2566). No differences were observed in naive CD4 T cells. In the CM compartment, CD38 expression was modestly lower in PCOS compared with endometriosis (*P *= 0.1095) and slightly lower than healthy controls (*P *= 0.3757), while healthy and endometriosis samples did not differ (*P *= 0.2586). Early-like EM CD4 T cells showed a gradual downward trend from healthy to endometriosis to PCOS (healthy vs endometriosis *P *= 0.2086; healthy vs PCOS *P *= 0.0697; endometriosis vs PCOS *P *= 0.3039), with a similar but less pronounced pattern in early EM cells. Terminal EM cells exhibited lower CD38 expression in PCOS than healthy individuals (*P *= 0.1151), whereas no differences were observed between endometriosis and PCOS (*P *= 0.2978) or between healthy and endometriosis (*P *= 0.9924). CD45RA^+^ TE cells did not differ across groups. Tregs displayed higher CD38 expression in endometriosis compared with healthy (*P *= 0.1543) and PCOS (*P *= 0.0492), while PCOS tended to be lower than healthy (*P *= 0.2510).

Although most of these comparisons did not reach *P *< 0.05 due to low replicates, the patterns observed across mitochondrial and activation markers indicate that menstrual blood in PCOS and endometriosis does not deviate in a single unified direction from healthy controls. Instead, each condition appears to show trends toward a distinctive immunological profile across multiple immune cell subsets. These pilot-level findings highlight the potential of this expanded 27-color panel to **detect biologically relevant, disease-specific variation in menstrual immune composition** across a broad range of cell types and offer insights into future work.

## Discussion

Our findings highlight the unique immune landscape of menstrual blood and demonstrate its potential as a powerful, noninvasive tool for exploring immune alterations in reproductive disorders such as endometriosis and PCOS. Using a novel high-dimensional immunoprofiling approach, we provide compelling evidence that the immune composition of MMCs is markedly distinct from that of matched PBMCs, and that these differences reflect both tissue-specific adaptations and disease-specific immune dysregulation.

In healthy individuals, the shift in NK cell phenotype toward a CD56^bright^CD16^dim^ profile, along with the enrichment of macrophages and dendritic cells in MMCs, suggests that menstrual blood carries hallmarks of mucosal and tissue-resident immunity. These findings are consistent with previous studies demonstrating that the menstrual blood reflects the endometrium which hosts a unique immune environment adapted to cyclical tissue remodeling, implantation, and defense against pathogens.^5^^,^  ^16–18^ The mitochondrial data further reinforce this tissue-resident profile: CD4 and CD8 T cells within MMCs displayed higher mitochondrial mass than their PBMC counterparts, suggesting increased reliance on oxidative phosphorylation and a metabolically active state, whereas monocytes showed reduced mitochondrial content. This metabolic dimension provides novel insight into how immune cells function within the reproductive tract.

Importantly, this study is among the first to integrate mitochondrial profiling into multiparameter flow cytometry of menstrual blood, adding a critical metabolic dimension to immunophenotyping. Immune metabolism is increasingly recognized as a central regulator of cell fate and function,^6^ and our finding that mitochondrial content varies significantly between PBMCs and MMCs—and across disease states—may open new avenues for understanding how metabolic dysregulation contributes to reproductive pathologies.

In addition to these baseline differences between MMCs and PBMCs, our data suggest that endometriosis and polycystic ovary syndrome (PCOS) each exhibit distinct menstrual immune signatures. Although the analysis is exploratory and based on a small cohort, several consistent patterns were evident. Mitochondrial mass tended to be lower in Tregs and CM CD4 T cells in PCOS compared with both healthy individuals and those with endometriosis, while plasmablasts displayed increased mitochondrial content in both reproductive disorders but with greater heterogeneity in endometriosis. cDC1 populations exhibited reduced mitochondrial mass in endometriosis relative to healthy controls, whereas PCOS samples showed broader variability. Together, these trends indicate that mitochondrial alterations in menstrual blood are not solely a feature of reproductive disease in general but instead differ by condition.

Activation-related markers also demonstrated disease-specific patterns. CD38 expression in total CD4 T cells and Tregs was higher in endometriosis compared with both healthy individuals and those with PCOS, while several CD4 EM populations showed a gradual downward shift in CD38 expression from healthy to endometriosis to PCOS. Although most comparisons did not reach conventional statistical significance, the directionality and consistency of these changes across multiple subsets support the presence of biologically relevant immune variation between disorders. Importantly, these examples demonstrate that menstrual blood can reveal nuanced immunological differences across a wide range of immune cell types, and reflects the chronic low-grade inflammation observed in PCOS^19–21^ and endometriosis.^22–24^

It should be noted that, although numerous significant or biologically meaningful differences were identified across the 546 features measured, it was not feasible to present graphical outputs for all parameters. Representative markers were therefore selected to illustrate key findings, with the complete dataset provided in the accompanying files for transparency and to enable further examination by the research community. These representative examples highlight the utility of menstrual blood immunoprofiling, while the full dataset demonstrates the breadth of accessible features for future exploratory or confirmatory studies.

This study has several limitations that should be considered when interpreting the findings. Menstrual blood samples were processed using dithiothreitol (DTT) to reduce viscosity. Although DTT can alter the expression of certain surface molecules—such as CD38^25^—this does not affect the ability to correctly identify major immune cell subsets, nor does it interfere with the overall immune landscape captured by our panel. The comparison between PBMCs and MMCs in this study was performed as a proof-of-principle demonstration to show that menstrual blood reflects a tissue-resident immune environment, and this broader interpretation is not compromised using DTT. As all menstrual samples (healthy, endometriosis, and PCOS) underwent identical processing, DTT does not confound disease-state comparisons. Nonetheless, alternative viscosity-reducing methods may be beneficial in future work to enable fully equivalent processing of PBMCs and MMCs for direct marker-level interpretation. Mitochondrial mass was used as an indicator for immunometabolic state; however, this measure does not directly reflect mitochondrial function, and functional assays will be required to confirm these observations. Diagnoses of endometriosis and PCOS were self-reported, and information on disease staging, or hormonal status was not available, which limits interpretation of disease-specific immune signatures. Finally, the study was exploratory and based on a small cohort (n = 4–5 per group), with high-dimensional data comprising both percentages and median fluorescence intensities. Formal multiple-comparison correction was not applied because of the small sample size, the high correlation between features, and the risk of eliminating biologically meaningful signals. Instead, raw *P* values were reported alongside an effect-size threshold of log_2_ fold-change ≥0.5 or ≤−0.5 to minimize trivial differences. These findings should therefore be considered hypothesis-generating and validated in larger, well-characterized cohorts.

Taken together, our data reinforce the concept that menstrual blood is not merely biological waste but a dynamic and information-rich medium that can reflect both physiological and pathological states of the reproductive tract. The ability to profile activation, differentiation, and metabolic features across numerous immune cell subsets provides a rich platform for future research. Larger studies will be essential to validate these findings, examine temporal variation across the menstrual cycle, and evaluate the potential of menstrual immune profiling as a diagnostic or prognostic tool for reproductive disorders such as endometriosis and PCOS.

## Supplementary Material

vlag013_Supplementary_Data

## Data Availability

All raw MFI and percentage values have been included in Tables S3–5.

## Abbreviations

peripheral blood mononuclear cells: PBMCs
menstrual blood mononuclear cells: MMCs
polycystic ovary syndrome: PCOS
