# Supplementary material for: Revealing the immune landscape of menstrual blood: unlocking insights into activation, exhaustion, and mitochondrial mass for reproductive health
Source: Immunohorizons. 2026 Mar 25;10(3):vlag013. doi: 10.1093/immhor/vlag013 (PMC13019135; doi:10.1093/immhor/vlag013)
Supplement: vlag013_Supplementary_Data [file vlag013_supplementary_data.zip › Supplemental Figures.pdf]

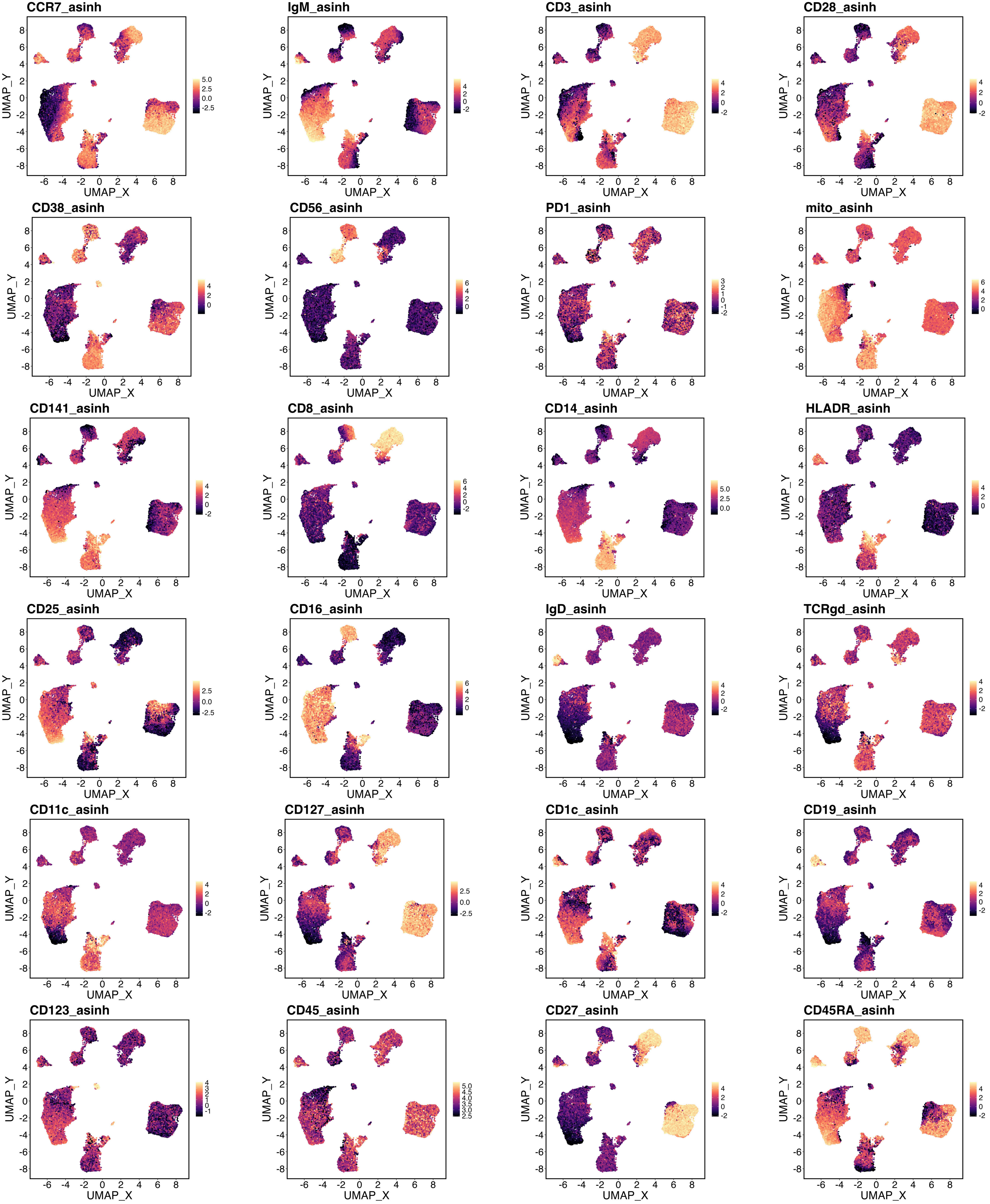

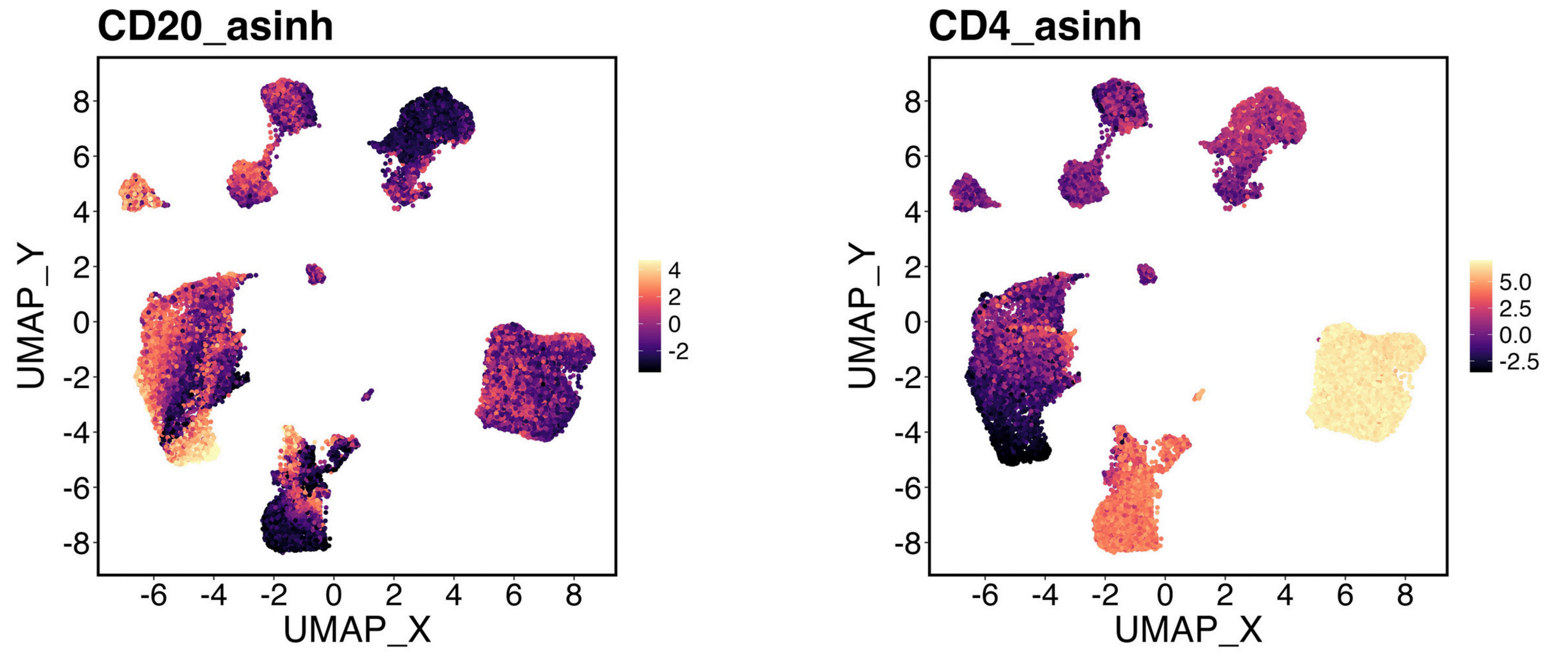

Figure S1: Pooled UMAPs of the clustering of the differential expression of each marker analysed for PBMCs vs MMCs.

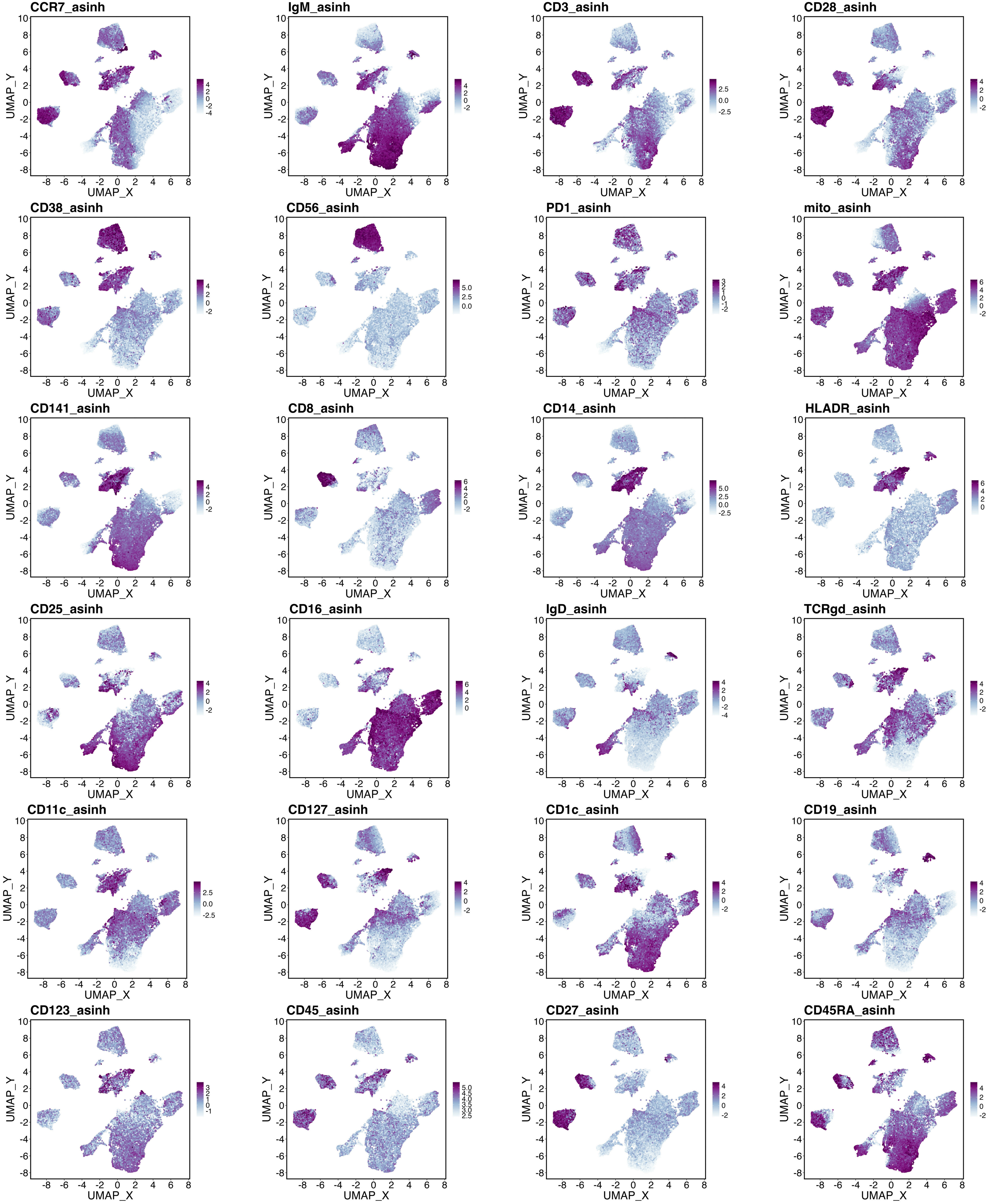

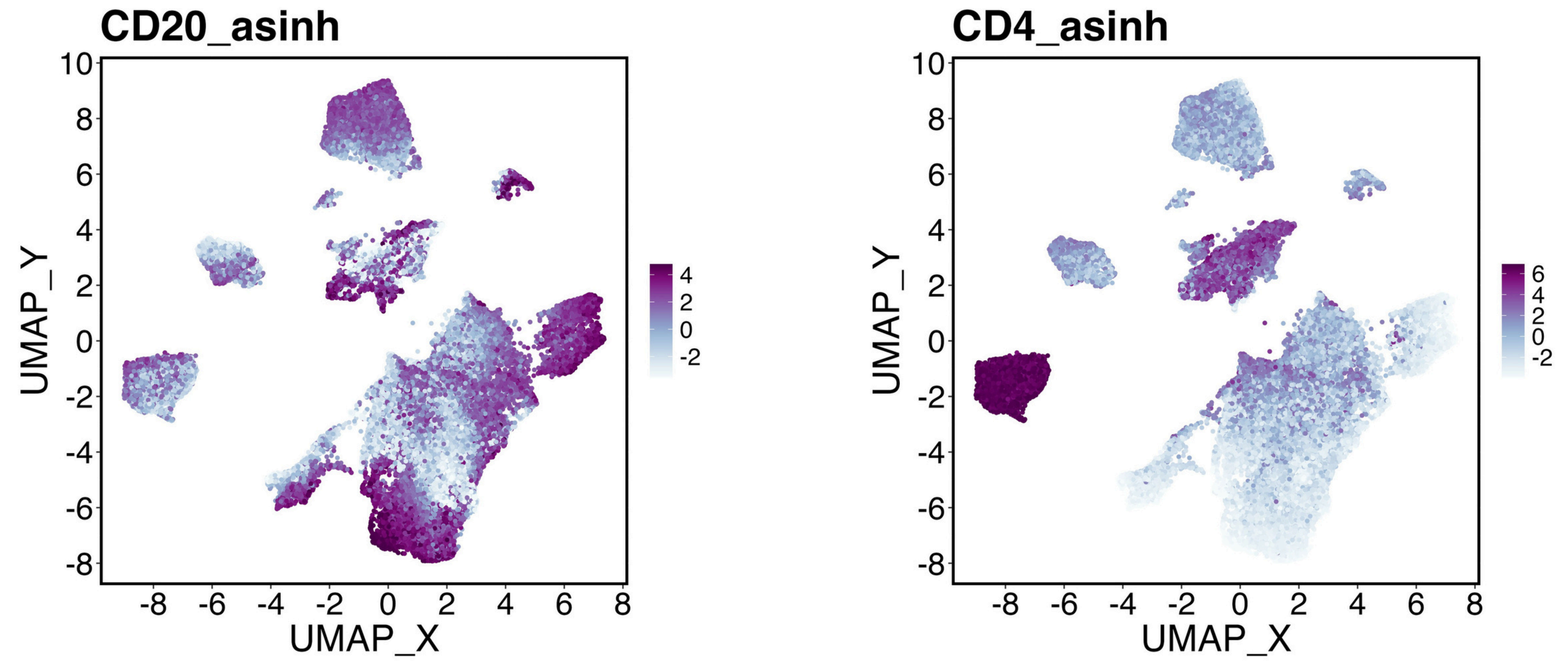

Figure S2: Pooled UMAPs of the clustering of the differential expression of each marker analysed for MMCs of healthy, PCOS and endometriosis.

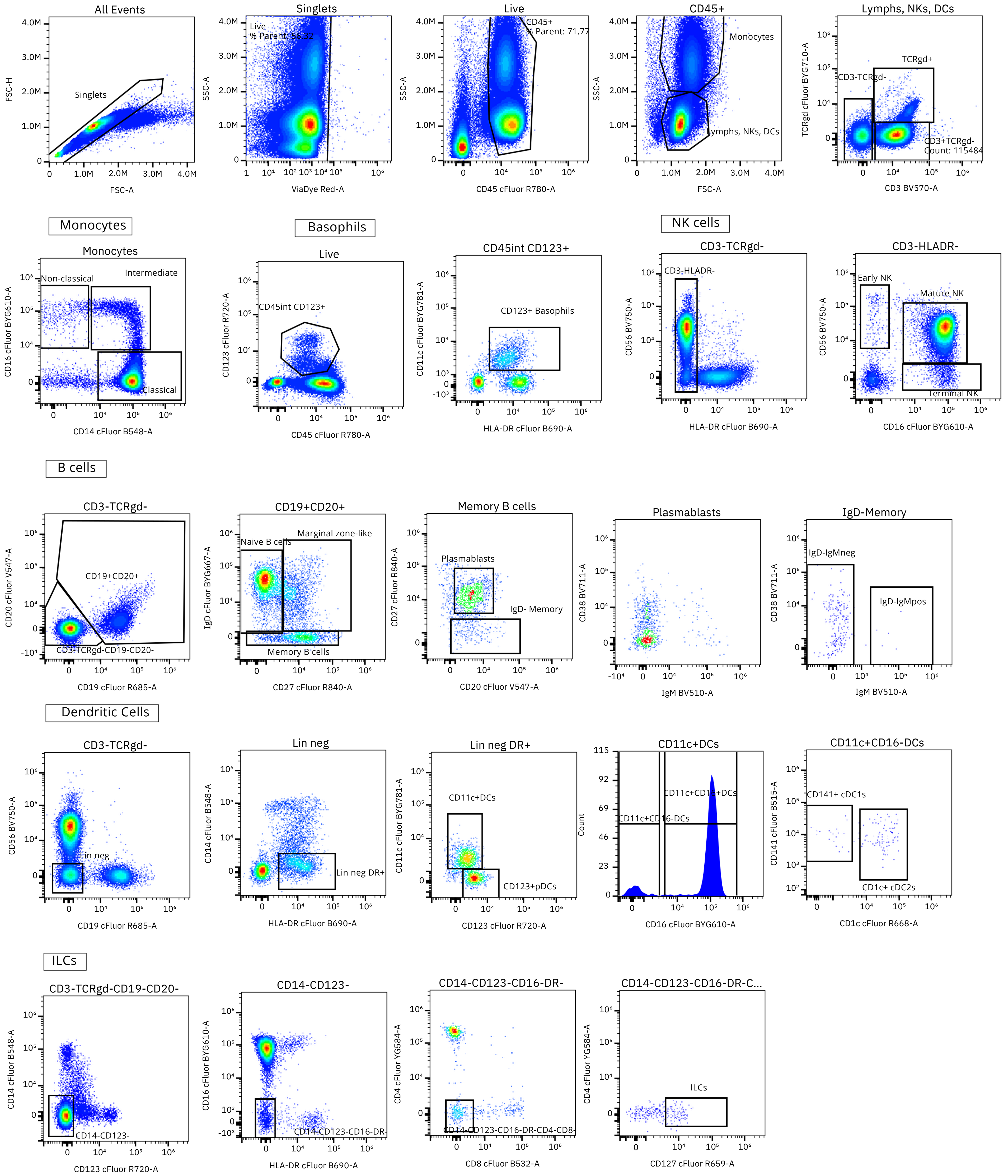

Figure S3: Gating strategy for identification of live CD45+ cells, and consequent monocytes, basophils, B cells, NK cells, DCs, and ILCs (with appropriate subsets).

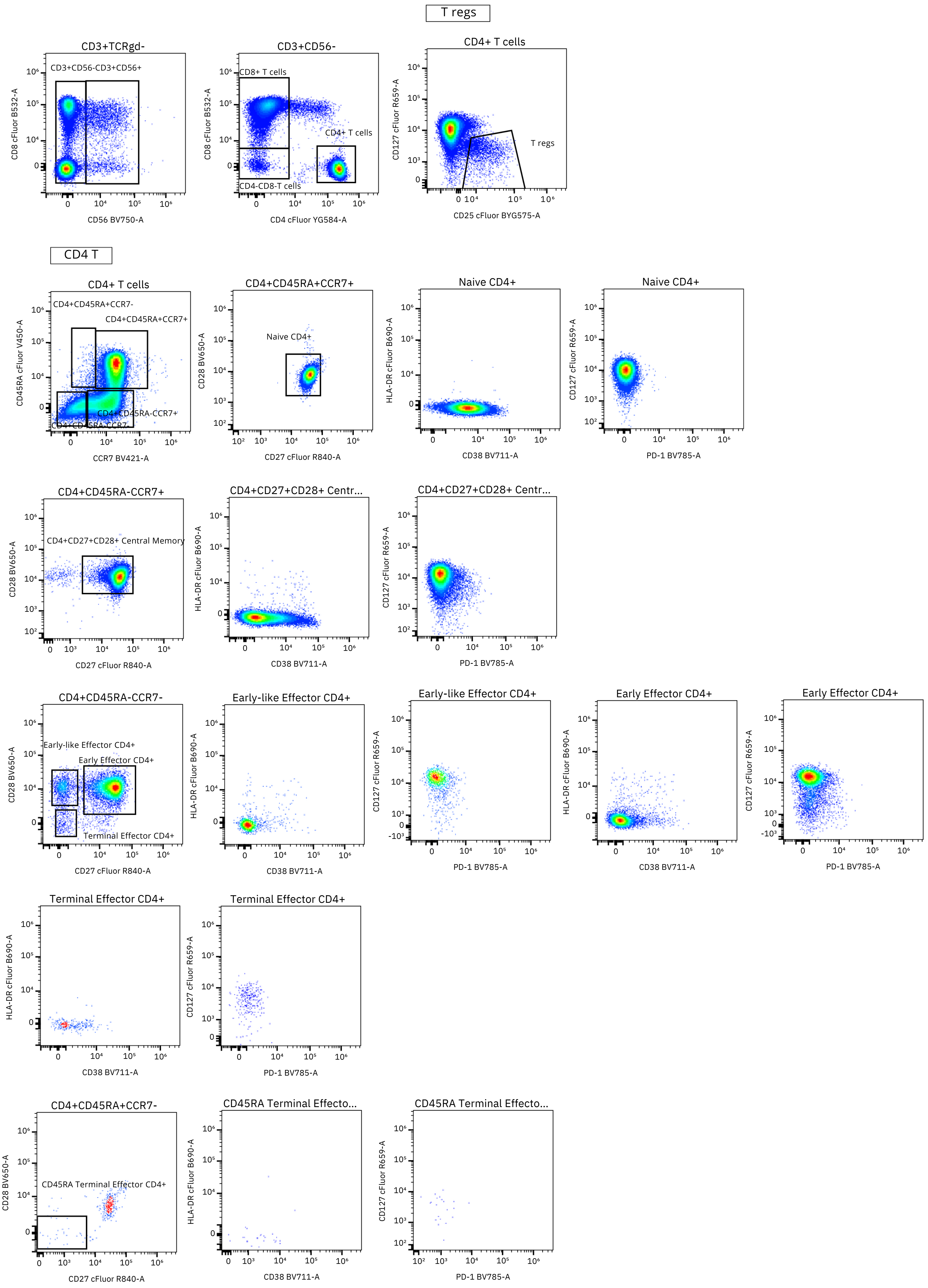

Figure S4: Gating strategy for subsequent identification of CD4 T cells and its subsets.

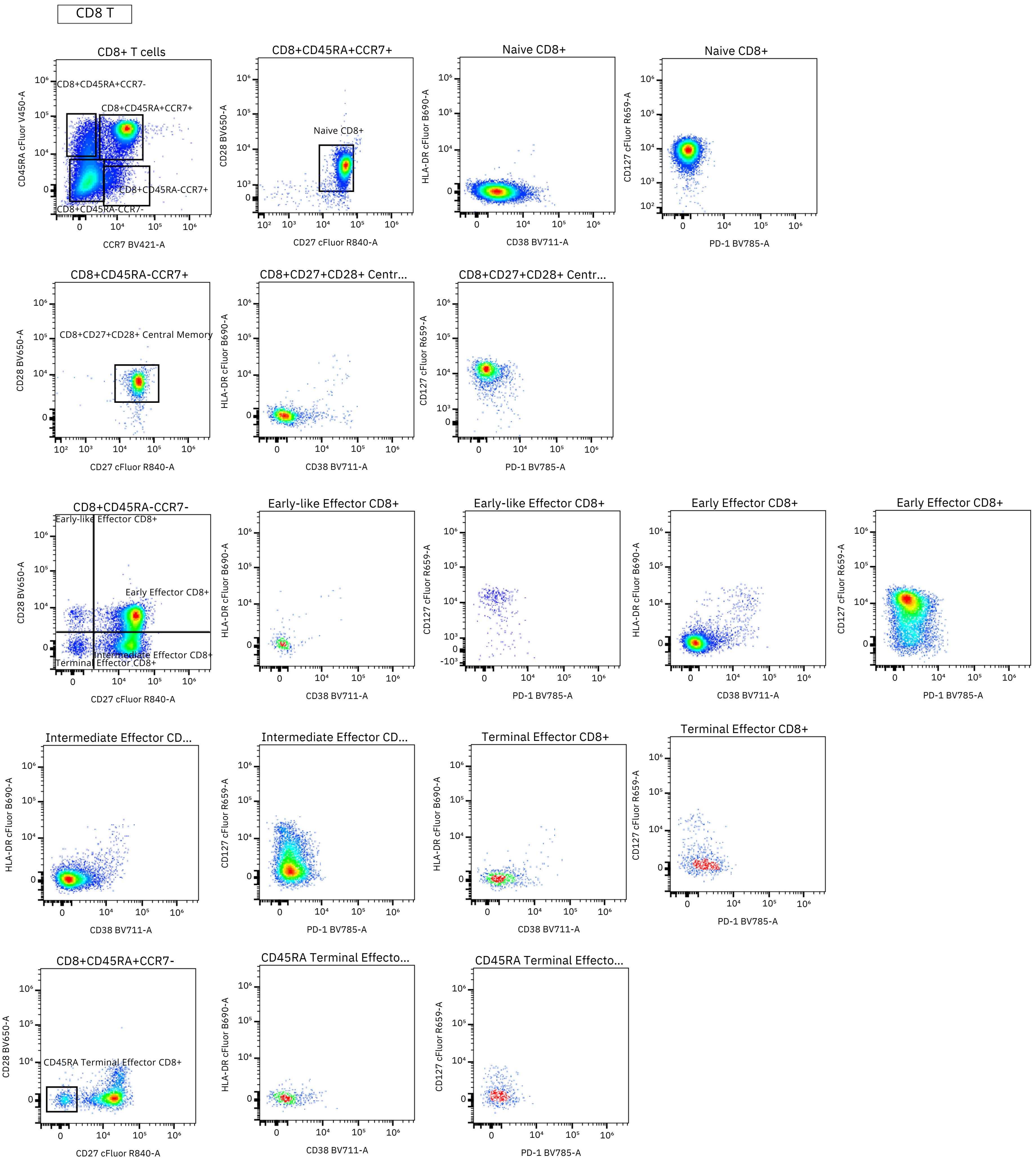

Figure S5: Gating strategy for subsequent identification of CD8 T cells and its subsets.
