# Supplementary material for: Revealing the immune landscape of menstrual blood: unlocking insights into activation, exhaustion, and mitochondrial mass for reproductive health
Source: Immunohorizons. 2026 Mar 25;10(3):vlag013. doi: 10.1093/immhor/vlag013 (PMC13019135; doi:10.1093/immhor/vlag013)
Supplement: vlag013_Supplementary_Data [file vlag013_supplementary_data.zip › Table S2.docx]

| **Cell type** | **Surface markers** |
| --- | --- |
| Basophils | CD45ᶦⁿᵗ, CD123⁺, HLA-DR⁻ |
| ILCs | CD3⁻, TCRγδ⁻, CD19⁻, CD20⁻, CD14⁻, CD123⁻, CD16⁻, HLA-DR⁻, CD4⁻, CD127⁺ |
| B cells | CD19⁺, CD20⁺ |
| Naïve | IgD⁺, CD27⁻ |
| Marginal Zone-like | IgD⁺, CD27⁺ |
| Memory | IgD⁻, CD27⁺ |
| Plasmablasts | CD20ˡᵒʷ, CD27ʰᶦᵍʰ |
| Monocytes | CD14⁺, HLA-DR⁺ |
| Classical | CD14ʰᶦᵍʰ, CD16⁻ |
| Intermediate | CD14ʰᶦᵍʰ, CD16⁺ |
| Non-classical | CD14ˡᵒʷ, CD16⁺ |
| Dendritic cells | Lin⁻, HLA-DR⁺ |
| pDCs | CD123⁺, CD11c⁻ |
| cDC1 | CD11c⁺, CD16⁻, CD1c⁻, CD141⁺ |
| cDC2 | CD11c⁺, CD16⁻, CD1c⁺ |
| NK cells | CD3⁻, TCRγδ⁻, CD56⁺ |
| Early NK | CD56⁺⁺, CD16⁻ |
| Mature NK | CD56⁺, CD16⁺ |
| Terminal NK | CD56ˡᵒʷ, CD16⁺ |
| NKT-like cells | CD3⁺, CD56⁺ |
| γδ T cells | CD3⁺, TCRγδ⁺ |
| Tregs | CD4⁺, CD25ʰᶦᵍʰ, CD127ˡᵒʷ, |
| CD4+ T cells | CD3⁺, CD4⁺, CD8⁻ |
| Naïve | CD45RA⁺, CCR7⁺ |
| Central memory | CD45RA⁻, CCR7⁺ |
| Effector memory | CD45RA⁻, CCR7⁺ |
| Early-like effector memory | CD27⁻, CD28⁺ |
| Early effector memory | CD27⁺, CD28⁺ |
| Intermediate effector memory | CD27⁺, CD28⁻ |
| Terminal effector memory | CD27⁻, CD28⁻ |
| CD45RA+ Terminal effector | CD45RA⁺, CCR7⁻ |
| CD8+ T cells | CD3⁺, CD8⁺, CD4⁻ |
| Naïve | CD45RA⁺, CCR7⁺ |
| Central memory | CD45RA⁻, CCR7⁺ |
| Effector memory | CD45RA⁻, CCR7⁺ |
| Early-like effector memory | CD27⁻, CD28⁺ |
| Early effector memory | CD27⁺, CD28⁺ |
| Intermediate effector memory | CD27⁺, CD28⁻ |
| Terminal effector memory | CD27⁻, CD28⁻ |
| CD45RA+ Terminal effector | CD45RA⁺, CCR7⁻ |
| CD4-CD8- T cells | CD3⁺, CD4⁻, CD8⁻ |

**Table S2:** Surface phenotype of cells identified.
