# Supplementary material for: Revealing the immune landscape of menstrual blood: unlocking insights into activation, exhaustion, and mitochondrial mass for reproductive health
Source: Immunohorizons. 2026 Mar 25;10(3):vlag013. doi: 10.1093/immhor/vlag013 (PMC13019135; doi:10.1093/immhor/vlag013)
Supplement: vlag013_Supplementary_Data [file vlag013_supplementary_data.zip › Table S1.docx]

**Table S1:** Antibody details.

| **Target** | **Fluorochrome** | **Clone** | **Catalogue Number** | **Manufacturer** |
| --- | --- | --- | --- | --- |
| CCR7 | Brilliant Violet 421™ | G043H7 | 353207 | BioLegend |
| CD45RA | cFluor® V450 | HI100 | R7-40002 | Cytek Biosciences |
| IgM | Brilliant Violet 510™ | MHM-88 | 314521 | BioLegend |
| CD20 | cFluor® V547 | 2H7 | R7-40002 | Cytek Biosciences |
| CD3 | Brilliant Violet 570™ | UCHT1 | 300435 | BioLegend |
| CD28 | Brilliant Violet 650™ | 28.2 | 302945 | BioLegend |
| CD38 | Brilliant Violet 711™ | HIT2 | 303527 | BioLegend |
| CD56 | Brilliant Violet 750™ | 5.1H11 | 362555 | BioLegend |
| PD-1 | Brilliant Violet 785™ | EH12.2H7 | 329929 | BioLegend |
| CD141 | cFluor® B515 | M80 | R7-40002 | Cytek Biosciences |
| CD8 | cFluor® B532 | SK1 | R7-40002 | Cytek Biosciences |
| CD14 | cFluor® B548 | 63D3 | R7-40002 | Cytek Biosciences |
| HLA-DR | cFluor® B690 | L243 | R7-40002 | Cytek Biosciences |
| CD25 | cFluor® BYG575 | BC96 | R7-40002 | Cytek Biosciences |
| CD4 | cFluor® YG584 | SK3 | R7-40002 | Cytek Biosciences |
| CD16 | cFluor® BYG610 | 3G8 | R7-40002 | Cytek Biosciences |
| IgD | cFluor® BYG667 | IA6-2 | R7-40002 | Cytek Biosciences |
| TCRγδ | cFluor® BYG710 | B1 | R7-40002 | Cytek Biosciences |
| CD11c | cFluor® BYG781 | 3.9 | R7-40002 | Cytek Biosciences |
| CD127 | cFluor® R659 | A019D5 | R7-40002 | Cytek Biosciences |
| CD1c | cFluor® R668 | L161 | R7-40002 | Cytek Biosciences |
| CD19 | cFluor® R685 | HIB19 | R7-40002 | Cytek Biosciences |
| CD123 | cFluor® R720 | 6H6 | R7-40002 | Cytek Biosciences |
| CD45 | cFluor® R780 | 2D1 | R7-40002 | Cytek Biosciences |
| CD27 | cFluor® R840 | QA17A18 | R7-40002 | Cytek Biosciences |
